# Combined glucocorticoids and cyclophosphamide in the treatment of Graves’ ophthalmopathy: a systematic review and meta-analysis

**DOI:** 10.1186/s12902-024-01545-0

**Published:** 2024-01-26

**Authors:** Qilang Xiang, Mengling Yang, Wenxuan Luo, Yuzi Cao, Shiquan Shuai, Xin Wei, Anji Xiong

**Affiliations:** 1 Department of Rheumatology and Immunology, Nanchong Central Hospital, The Affiliated Nanchong Central Hospital of North Sichuan Medical College/Nanchong Hospital of Beijing Anzhen Hospital Capital Medical University, Nanchong, China; 2 Department of Gastroenterology, The Affiliated Hospital of Southwest Medical University, Luzhou, China; 3 Department of Ophthalmology, West China Hospital, Sichuan University, Chengdu, China; 4 School of Medicine, Chongqing University, Chongqing, China

**Keywords:** Graves ophthalmopathy, Glucocorticoids, Cyclophosphamide, Meta-analysis, Systematic review

## Abstract

**Purpose:**

To evaluate the efficacy and safety of combined glucocorticoids (GCs) and cyclophosphamide (CYC) treatment in Graves’ ophthalmopathy (GO).

**Methods:**

We searched PubMed, Embase, Cochrane Library, and four Chinese databases (Chinese National Knowledge Infrastructure (CNKI), China Science and Technology Journal Database (VIP), WanFang, and SinoMed) for any published randomized controlled trials (RCTs) produced from inception to December 1, 2023. Articles obtained using appropriate keywords were selected independently by two reviewers according to the established inclusion and exclusion criteria.

**Findings:**

We retrieved 1120 records which were eventually reduced to 13 RCTs which were then included in this evaluation. Pooled results indicated that the experimental group (CYC/GCs) showed a higher response rate than control group (GCs or negative control) (RR 1.27; 95% confidence interval 1.19 to 1.37). The subgroup analysis showed that the difference in response rates among treatment protocols (CYC/P, CYC/MPS, CYC/DEX) was not statistically significant (*p* = 0.23).

**Implications:**

The combination of GCs and CYC could be recommended as a therapeutic option for GO, especially in patients who experience recurrence after a withdrawal GCs, have a poor response to GCs, or cannot obtain monoclonal antibody agents for various reasons.

**Supplementary Information:**

The online version contains supplementary material available at 10.1186/s12902-024-01545-0.

## Introduction

Graves’ ophthalmopathy (GO), also called thyroid-associated ophthalmopathy (TAO), is mainly characterized by proptosis, upper eyelid retraction, edema, and diplopia and is described as an ocular autoimmune disorder with complicated pathogenesis [1, 2]. Increased adipose tissue and enlarged extraocular muscle within the ocular orbit are the primary pathological changes associated with GO [3], which causes increased pressure within the bony cavity ultimately inducing a cascade of clinical symptoms [1, 4]. Increasing evidence suggests that orbital fibroblasts are critical effector cells during the pathogenesis of GO [4, 5]. Current evidence suggests that the thyrotropin and insulin-like growth factor receptors (IGF-1R), both expressed on the surface of the orbital fibroblasts, are the primary autoantigens in GO [1]. The immune systems of patients with GO are known to be hyperactivated with an increased proliferation of autoreactive B cells that produce a large number of autoantibodies [1]. In addition, stimulation with anti-thyrotropin-receptor antibodies induces a subgroup of orbital fibroblasts to differentiate into adipocytes [1, 6, 7]. IGF-1R binding by autoantibodies results in the secretion of interleukin-16, leading to the recruitment of T cells and other mononuclear immune cells into the ocular orbit [1, 8]. Orbital fibroblasts can also be activated by these T cells via the CD40–CD154 bridges, inducing interleukin-1 (IL-1) secretion [1, 4]. Stimulation with IL-1, TNF, and IFN-γ induces orbital fibroblast-based production of hyaluronan which then accumulates between the intact extraocular muscle fibers [1]. The cell-surface marker, Thy-1, is expressed in a population of fibroblasts, which can differentiate into myofibroblasts upon stimulation with transforming growth factor (TGF)-β [1, 4]. These responses act together to facilitate GO pathogenesis which can be summarized as an autoimmune reaction that leads to increases in the inflammatory response and tissue changes in the orbit, eventually resulting in expanded adipose tissue, extraocular muscle enlargement, and fibrosis [1].

Glucocorticoids (GCs) are strong immunosuppressive and anti-inflammatory drugs considered the first-line treatment for GO [9–11]. GCs inhibit both the proliferation and function of lymphocytes by inhibiting the release of lymphokines and cytokines [12–14], thereby exerting an immunosuppressive and anti-inflammatory effect. However, in the case of more severe autoimmune diseases, including those that cause organ damage (such as GO), it is not unusual to combine GCs with other potent immunosuppressive drugs. Cyclophosphamide (CYC), a good option to combination therapy, has been used in clinical practice for over 60 years and remains one of the most useful anticancer and immunosuppressive agents [15, 16]. CYC inhibits immune responses by directly inhibiting the proliferation of lymphocytes and has significant effects on the treatment of various autoimmune diseases [15, 17, 18]. The combination of GCs and CYC is commonly applied in the clinical treatment of GO in China and its curative effect has proven remarkable [19, 20]. However, until December 1, 2023, there was no published meta-analysis have assessed the efficacy of this combination in the treatment of GO. Therefore, we conducted a systematic review and meta-analysis of published randomized clinical trial (RCT) data to assess the efficacy and safety of combined GCs/CYC to treat GO.

## Methods

### Data sources and search strategies

Our study relied on data from PubMed, Embase, the Cochrane Library, Google Scholar, and three Chinese databases (the Chinese National Knowledge Infrastructure (CNKI), China Science and Technology Journal Database (VIP), WanFang, and SinoMed). We identified studies of interest published from inception to December 1, 2023 and isolated all the RCTs. The detailed search strategy is presented in Supplementary Appendix 1.

### Inclusion and exclusion criteria

Our database search yielded 1120 hits, which were then narrowed to only the RCTs meeting the following criteria: (1) patients were diagnosed with GO, with no age limit; (2) All patients were routinely treated with antithyroid drugs or levothyroxine; (3) in experimental groups, GCs and CYC were given as main intervention measure in the treatment, regardless of cumulative dose or route of administration; (4) in control group, patients were treated with GCs alone or with negative controls; (5) there were no significant differences in the general clinical characteristics between these two groups (*P* > 0.05); and (6) evidence of at least one of the following four outcomes (response rate, adverse events, clinical activity score (CAS), and proptosis). The following exclusion criteria were applied: (1) Studies were failing to comply with these inclusion criteria (2) studies had duplicate data or repeat analysis; (3) studies were case reports, reviews, letters, or conference abstracts without full text. The language of the articles was limited to Chinese and English.

### Study selection and data extraction

All publications were reviewed by two independent researchers (QX and MY) before their inclusion in this study. These reviewers evaluated the eligibility of the potential titles and abstracts and then rigorously reassessed the full text following their initial selection using the same inclusion criteria described above. Relevant data were then extracted by two different researchers (YC and SS) as described by the predesigned review form. They carefully extracted the following information from each study: the first author’s name, year of publication, number of patients, route of administration, and outcomes (response rate, adverse events, CAS, and proptosis). We resolved disagreements by discussing these factors and then referring to a third reviewer where we could not reach a consensus.

### Quality assessment

Each of the included studies was then evaluated for quality by two other researchers (XW and AX) using the Cochrane Collaboration risk of bias tool [21] for the following six aspects: random sequence generation, allocation concealment, blinding of participants and personnel, incomplete outcome data, selective reporting, and other biases. We graded each item as “low,” “high,” or “unclear” risks of bias. The GRADE framework was used to grade the evidence quality of the outcomes. (Supplementary Appendix 4).

### Statistical analysis

Of the 13 RCTs, only one study [22] included reduction of proptosis as its outcome. The remaining 12 RCTs either did not evaluate or only reported start and end point data for their continuous variables (i.e., proptosis, CAS) as the mean ± SD. Given that most of the continuous variable data was incomplete, we chose to focus our data analysis on the dichotomous variables (number of patients with response). All data analysis were performed using R software (R version 4.2.1) and we used the risk ratio and proportion and their associated 95% confidence intervals to assess outcomes. To enhance the methods, some previously published meta-analysis articles were referred [23, 24]. All *P* values of less than 0.01 were considered statistically significant and heterogeneity was assessed using the I^2^ test. If significant heterogeneity was not present (I^2^ < 50%), we used fixed effects models to pool outcomes and if there was significant heterogeneity, we pooled outcomes using random effects models (I^2^ ≥ 50%). Sensitivity analysis was also performed by removing each study in turn and publication bias was evaluated using funnel plots and the Egger test. When publication bias occurred, the “cut-and-fill method” was performed to adjust publication bias.

## Results

### Study selection and characteristics

**Fig. 1:**
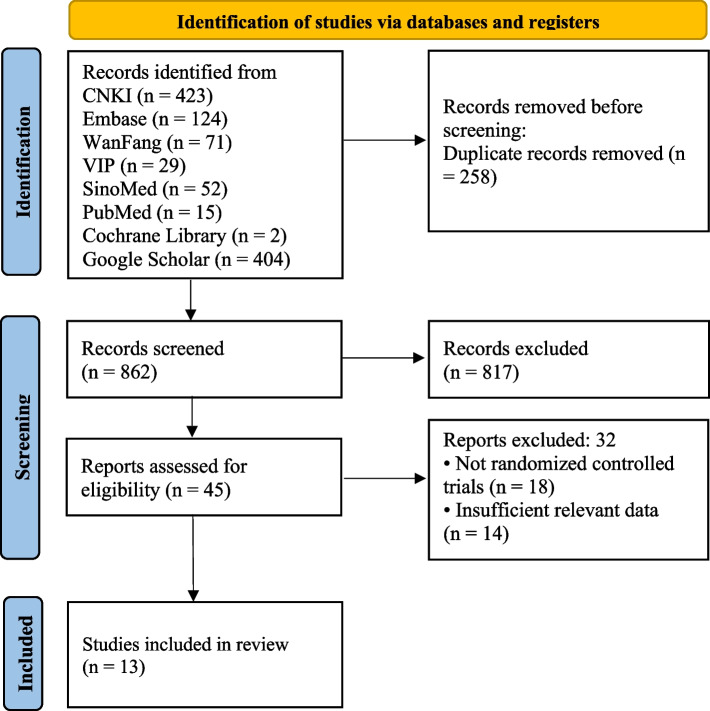
Flow diagram for literature search and study selection

Our initial retrieval strategy identified 1120 records, which included 13 eligible trials [22, 25–36] that were then used in the final meta-analysis. Figure 1 summarizes our literature search and study selection process. All 13 RCTs included in this study were conducted in China and published in Chinese between 2004 and 2022. A total of 932 patients were included in the meta-analysis, with 472 patients assigned to the experimental group and 460 patients randomly assigned to the control groups. Of the 13 studies included in this analysis, all 13 reported the number of patients with responses, five studies reported the adverse reactions, and one reported a reduction in proptosis [22]. Treatment duration varied from 1 to 3 months and information was not available for one study [29]. In all 13 studies, interventions of control groups were GCs in 7 studies, and 6 studies had a negative control. Among the combined treatment protocols of experimental groups, 2 studies used CYC/dexamethasone (DEX), 8 studies used CYC/methylprednisolone (MPS), and 3 studies used CYC/prednisone (P). The characteristics of each of the included studies are summarized in Table 1 and the detailed information on treatment protocols and adverse reactions of each included study is summarized in Table 2.

**Table 1:** Characteristics of included studies

Study	Sample size (E/C)	Experimental groups (CYC/GCs)	Cumulative CYC/GCs dose	Control groups	Treatment for the thyroid disorder	Treatment duration	Number of patients with response (E/C)	Criteria for response
Shi 2004 [25]	53/52	CYC/DEX	0.1 g/0.05 g	GCs (DEX)	ATD	10 weeks	29/25	Reduction of proptosis ≥ 2mm
Liu 2005 [26]	30/30	CYC/MPS	1.2 g/3 g	GCs (MPS)	ATD	1 month	29/24	Decline of CAS ≥ 2
Zhang 2005 [22]	30/30	CYC/MPS	2.4 g/6 g	Negative control	ATD	1 month	30/2	Reduction of proptosis > 2mm
Ruan 2006 [27]	54/51	CYC/DEX	0.5 g/0.05 g	GCs (DEX)	ATD	10 weeks	35/30	Reduction of proptosis > 2mm
Tang 2006 [28]	30/27	CYC/P	1.6 g/0.96 g	GCs (P)	ATD	1 month	29/11	Reduction of proptosis > 2mm
Su 2014 [29]	24/24	CYC/MPS	NA/NA	Negative control	Thiamazole	NA	23/19	NA
Dai 2015 [30]	22/22	CYC/MPS	6 g/3 g	GCs (P)	ATD	3 months	19/14	Reduction of proptosis ≥ 2mm
Wang 2015 [31]	40/40	CYC/P	4.48 g/1.16 g	Negative control	ATD	4 months	36/19	Reduction of proptosis ≥ 2mm
Wen 2016 [32]	46/46	CYC/MPS	1.8 g/4.5 g	Negative control	ATD/Levothyroxine	3 weeks	40/32	Reduction of proptosis ≥ 2mm and decline of CAS ≥ 2
Gao 2018 [33]	29/29	CYC/MPS	1.8g/4.5 g	Negative control	ATD/Levothyroxine	3 weeks	25/18	Reduction of proptosis ≥ 2mm and decline of CAS ≥ 2
Liang 2020 [34]	40/39	CYC/MPS	3 g/0.96 g	GCs (MPS)	ATD	3 months	39/31	NA
Cajal 2020 [35]	40/40	CYC/MPS	2.7g/5.4 g	GCs (MPS)	ATD	3 months	38/31	NA
Wang 2021 [36]	34/30	CYC/P	5.6 g/2.73 g	Negative control	Thiamazole	3 months	31/23	Reduction of proptosis > 2mm

*Abbreviations: CYC* cyclophosphamide, *GCs* glucocorticoids, *MPS* methylprednisolone, *DEX* dexamethasone, *P* prednisone, *ATD* antithyroid drug, *NA* not available

**Table 2:** Detailed information about treatment protocol and adverse reactions of included studies

Study	Treatment protocol		Adverse reactions	
	**CYC/GCs**	**Control group**	**CYC/GCs**	**Control group**
Shi 2004 [25]	Retrobulbar injection of DEX 5 mg and CYC 10 mg every week for 10 weeks	Retrobulbar injection of DEX 5 mg every week for 10 weeks	Conjunctival edema; retrobulbar hemorrhage; ptosis	Conjunctival edema; retrobulbar hemorrhage; ptosis
Liu 2005 [26]	Intravenous MPS 0.5g and CYC 0.2g every week for 4 weeks	Intravenous MPS 0.5g every week for 4 weeks	GCs-related adverse reactions (4 cases); electrolyte disorders (1 case)	GCs-related adverse reactions (8 cases); electrolyte disorders (3 cases)
Zhang 2005 [22]	Intravenous MPS 1.5g and CYC 0.6g every week for 4 weeks	Negative control	Mild euphoria (3 cases); insomnia (3 cases); stomach discomfort (3 cases)	Mild euphoria (4 cases); insomnia (4 cases); hypokalemia (5 cases); thrombocytopenia (3 cases)
Ruan 2006 [27]	Retrobulbar injection of DEX 5 mg and CYC 50 mg every week for 10 weeks	Retrobulbar injection of DEX 5 mg every week for 10 weeks	Conjunctival edema; retrobulbar hemorrhage; ptosis	Conjunctival edema; retrobulbar hemorrhage; ptosis
Tang 2006 [28]	Intravenous CYC 0.8g every week in the first two weeks, oral MPS 0.24g every week in the next two weeks	Negative control	NA	NA
Su 2014 [29]	Intravenous MPS 0.48g and oral CYC 0.2g each day	Negative control	NA	NA
Dai 2015 [30]	Intravenous MPS 1g/day for 3 days, then intravenous CYC 2g every month for 3 months	Oral P 60mg/day for 3 months	Elevated blood pressure (3 cases); elevated blood glucose (2 cases); weight gain (6 cases); severe infections (4 cases)	Elevated blood pressure (1 case); elevated blood glucose (5 cases); weight gain (10 cases); severe infections (1 case)
Wang 2015 [31]	In the first 8 weeks, oral P 60mg/day, and then reducing P dose 15mg/day every week. At 9th week, oral CYC 80mg/day for 8 weeks	Negative control	NA	NA
Wen 2016 [32]	Intravenous MPS 1.5g and CYC 0.6g every week for 3 weeks	Negative control	NA	NA
Gao 2018 [33]	Intravenous MPS 1.5g and CYC 0.6g every week for 3 weeks	Negative control	Mild euphoria (3 cases); stomach discomfort (3 cases); insomnia (2 cases)	Nil
Liang 2020 [34]	Oral CYC 100mg/day for 3 months, intravenous MPS 80mg/week for 3 months	Intravenous MPS 80mg/week for 3 months	Dyspepsia (1 case); stomatitis (1 case); alopecia (1 case)	Nausea (1 case); dyspepsia (1 case)
Cajal 2020 [35]	Intravenous MPS 0.6g and CYC 0.3g three times a month for 3 months	Intravenous MPS 1g three times a month for 3 months	NA	NA
Wang 2021 [36]	In the first 5 weeks, oral P 60mg/day, and then reducing P dose 15mg/day every week. At 6th week, oral CYC 100mg/day for 8 weeks	Negative control	NA	NA

*Abbreviations: CYC* cyclophosphamide, *GCs* glucocorticoids, *MPS* methylprednisolone, *DEX* dexamethasone, *P* prednisone, *NA* not available

### Risk of bias assessment

**Fig. 2:**
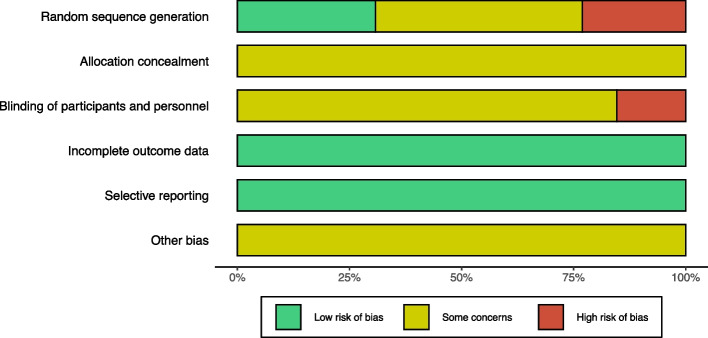
Risk of bias graph (By R language 4.2.1)

The Cochrane risk of bias assessment tool was used to assess the quality of the included studies. The included RCTs seemed to have a low to moderate risk of bias with most providing some details on allocation concealment and blinding. The risk of bias evaluations for each of these RCTs is described in Fig. 2 and the details of each risk of bias are shown in Supplementary Appendix 5.

### Response rate

**Fig. 3:**
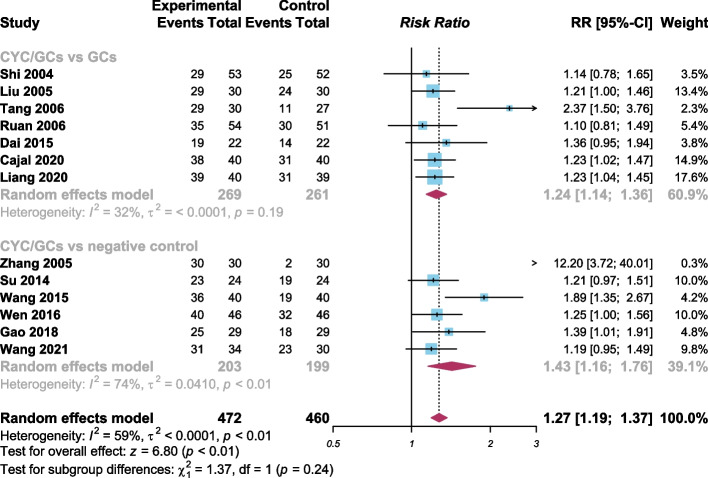
Forest plot of meta-analysis of response rate (CYC = cyclophosphamide; GCs = glucocorticoids; by R language 4.2.1)

All 13 studies reported response rate. The criteria for response differed between these studies, reduction of proptosis ≥ 2mm or > 2mm, and decline of CAS ≥ 2 were considered as responses. The total response rates for the experimental and control groups were 85.4% and 60.7%, respectively with the experimental group demonstrating a higher response rate than the control group (RR 1.27, 95% confidence interval 1.19 to 1.37, Fig. 3), with some heterogeneity (I^2^ = 59%, random effects model). We then explored this heterogeneity using a sensitivity analysis which was performed by removing each study in sequence. This sensitivity analysis showed that heterogeneity decreased (I^2^ = 0%) after excluding the Zhang (2005) study [22].

A subgroup analysis was then performed with each subgroup separated based on the intervention of their control group. From the subgroup CYC/GCs vs negative control, the experimental group showed a higher response rate than the control group (RR 1.43, 95% confidence interval 1.16 to 1.76, Fig. 3). From the subgroup CYC/GCs vs GCs, the experimental group also showed a higher response rate than the control group (RR 1.24, 95% confidence interval 1.14 to 1.36, Fig. 3). Subgroup analysis indicated that the combined treatment CYC/GCs was effective and even more effective than GCs in the treatment of GO.

**Fig. 4:**
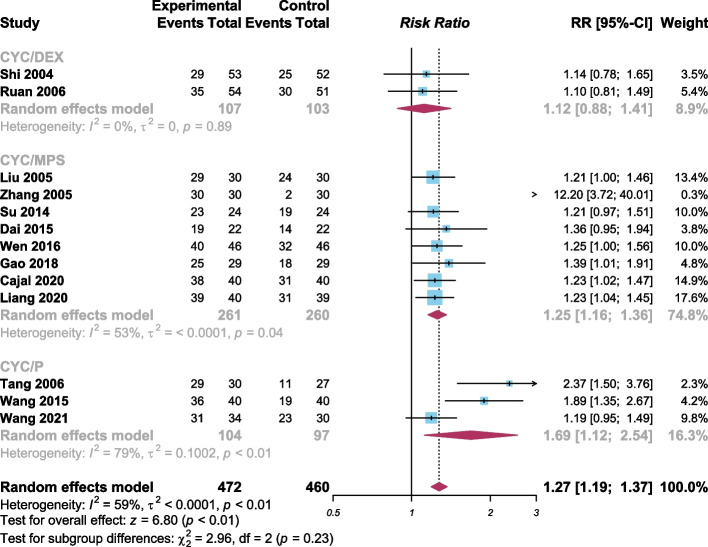
Forest plot of subgroup meta-analysis according to treatment protocols (CYC = cyclophosphamide; DEX = dexamethasone; MPS = methylprednisolone; P = prednisone; by R language 4.2.1)

For further exploration of the response rate of the combined treatment (CYC/GCs) and a definition of which combined treatment protocol was optimal in treating GO, we performed another subgroup analysis according to the combined CYC/GCs treatment protocols. The separate result of the subgroup (CYC/DEX) showed no statistically significant effect of the (CYC/DEX) treatment protocol (RR 1.12, 95% confidence interval 0.88 to 1.41, Fig. 4). Between another two subgroups, (CYC/P) had a greater response rate (RR = 1.69) compared with (CYC/MPS) (RR = 1.25), however, not statistically significant (*p* = 0.23, Fig. 4).

### Publication bias

Publication bias was investigated using funnel plots and the Egger test. The funnel plot was reasonably symmetrical aside from 2 outliers (Supplementary Appendix 6), and the Egger test (t = 3.94, *P* = 0.0023) suggested a risk of publication bias. Thus, the “cut-and-fill method” was performed to adjust the publication bias. After the “cut-and-fill method”, one missing study was added, and the response rate had been estimated (RR 1.26, 95% confidence interval 1.18 to 1.36, Supplementary Appendix 7).

## Discussion

In the early stages of the pathogenesis of GO, there are a large number of inflammatory reactions that can only be controlled by the powerful anti-inflammatory effects of the GCs. However, the abnormal autoimmune responses causing the ocular lesions in GO cannot be corrected by treatment with GCs, which serves only to reduce the immune response in these patients. If GCs treatment did not need to be reduced over time, as recommended in the guidelines, recurrence would probably not occur. However, in clinical practice, recurrence often occurs during GCs reduction or following GCs withdrawal [3, 37–39]. Additionally, if patients with GO receive long-term treatment with moderate to high doses of GCs designed to prevent recurrence, they almost inevitably develop a series of adverse reactions including glaucoma, severe hypertension, gastrointestinal bleeding, osteoporosis, adrenal gland atrophy, and uncontrolled diabetes, among others [13, 37], which would outweigh the benefits of continued therapy. Moreover, for patients with GO with contraindications such as hepatic dysfunction, cardiovascular morbidity, diabetes, or hypertension, GCs therapy could be even more undesirable. Therefore, therapeutic management of autoimmune diseases such as GO require the use of other immunosuppressive agents. Since the eye is an essential organ, it is even more essential to identify a rapid acting, effective immunosuppressing therapy (CYC) designed to prevent irreversible fibrosis in these patients. Furthermore, CYC can facilitate the successful withdrawal of GCs from patients with GO. This explains why several studies have proposed the combination of GCs and CYC in the initial phases of GO.

At present, many treatment options for GO exist but GCs remain the preferred treatment option [11]. Combination therapeutic regimens include GCs in combination with immunosuppressants such as cyclosporine or azathioprine, or orbital radiotherapy [11]. In addition, monoclonal antibodies such as rituximab (CD-20 antibody), tocilizumab (IL-6 receptor antibody), and teprotumumab (IGF-1 receptor antibody) are also recommended therapies for GO [11]. However, there are certain limitations to the treatment options above. For patients treated with GC monotherapy, the relapse rate during GC dose tapering or after GC withdrawal can be remarkable [38]. Cyclosporine and azathioprine have a relatively slow onset of action and are therefore not suitable for relapsing patients with GO whose symptoms need to be controlled rapidly. The combination of orbital radiotherapy and GCs is recommended based on expert opinion only and there is insufficient evidence in support of the treatment [11]. The main therapeutic mechanism of radiotherapy is the inhibition of the proliferation of effector cells, which is similar to CYC. Teprotumumab was approved by the FDA to treat GO in 2020 [40]; the benefits of rituximab and tocilizumab in the treatment of GO still require more clinical trials and evidence-based medical research. These monoclonal antibody agents may be not effective for all patients because of the single therapeutic targets [41] and they are costly and thus not affordable for all patients. In comparison, CYC is cheaper and available, with a fast onset of action, and the efficacy of CYC/GCs is supported by the results of our study. Therefore, for patients with GO who are prone to relapse after GC withdrawal, have difficulty in deriving therapeutic benefits from GCs, and in those who cannot obtain monoclonal antibody agents for various reasons, we believe that combined CYC/GCs treatment could be a therapeutic option.

Unfortunately, as CYC is rarely administrated by endocrinologists and ophthalmologists, practitioners may not be familiar with the characteristics of CYC. As rheumatologists, we have accumulated extensive experience in the use of CYC for a range of autoimmune diseases. It must be emphasized that CYC may cause significant cytotoxic effects such as infections, bone marrow suppression, impairment of the reproductive system, hemorrhagic cystitis, and pulmonary fibrosis [15]. However, these adverse reactions are often dose-related [15], making it critical to determine the minimum dose of CYC needed to prevent recurrence in these patients. Measures need to be undertaken to maximize the therapeutic efficacy of CYC and minimize undesirable reactions as much as possible. First, patients who present with CYC-associated contraindications, such as bone marrow suppression or lymphopenia, should not be considered for CYC treatment; it is also important to eliminate infections before the initiation of CYC treatment. Second, close observation of patients is essential, and those showing a trend toward adverse events should undergo adjustment of medication. Considering the heterogeneity in patient response to CYC, an individual optimal CYC dose in combined treatment should be gradually explored if patients can tolerate CYC and do not exhibit serious adverse events.

However, we acknowledge that there were some limitations to our study. First, because data for some of the continuous variables were incomplete, we were forced to complete our data analysis only using the dichotomous variables, limiting precision. Second, out of the limitations in safety-related data of included studies, our assessment on the safety aspect was unfortunately not enough. Third, the total number of studies and patients included in the meta-analysis for safety was small, so the quality of the evidence was not high. Fourth, most of these RCTs did not provide systematic follow-up data, which makes it difficult to assess the prognosis of GO after treatment. Fifth, all of the included patients were Chinese which means that the efficacy and safety of this combined therapeutic approach remains unknown for other ethnic groups. Sixth, among the included studies, only the Cajal (2020) study followed a treatment protocol consistent with the recommendations of international scientific societies. Seventh, sensitivity analysis suggested the Zhang (2005) study may be the source of the heterogeneity. It is possible that more severe and complex patients were included in this study, which resulted in a low response rate in the negative control. In addition, publication bias exists accordingly to the Egger test.

## Conclusion

Our systematic review and meta-analysis are the first to assess the efficacy of the combined regimen of CYC/GCs in the treatment of patients with GO. The combination of GCs and CYC could be a viable therapeutic option in GO especially in patients who have a poor response to GCs treatment, who relapse after GC withdrawal, or who cannot obtain monoclonal antibody agents. More clinical studies are required to determine the optimal medication regimen for the combination of GCs and CYC in the treatment of GO.

### Supplementary Information

**Additional file 1: Appendix 1.** Search strategy. **Appendix 2.** The kappa score between the researchers. **Appendix 3.** PRISMA checklist. **Appendix 4.** The quality of evidence using the GRADE framework. **Appendix 5.** Assessment of study quality. **Appendix 6.** Funnel plot of meta-analysis of response rate (By R language 4.2.1). **Appendix 7.** Forest plot of meta-analysis of response rate using the “cut-and-fill method” (By R language 4.2.1).

## Data Availability

The original contributions presented in the study are included in the article. Further inquiries can be directed to the corresponding authors.
