# Supplementary material for: Combined glucocorticoids and cyclophosphamide in the treatment of Graves’ ophthalmopathy: a systematic review and meta-analysis
Source: BMC Endocr Disord. 2024 Jan 26;24:12. doi: 10.1186/s12902-024-01545-0 (PMC10811849; doi:10.1186/s12902-024-01545-0)
Supplement: Supplementary file 1 — Additional file 1: Appendix 1. Search strategy. Appendix 2. The kappa score between the researchers. Appendix 3. PRISMA checklist. Appendix 4. The quality of evidence using the GRADE framework. Appendix 5. Assessment of study quality. Appendix 6. Funnel plot of meta-analysis of response rate (By R language 4.2.1). Appendix 7. Forest plot of meta-analysis of response rate using the “cut-and-fill method” (By R language 4.2.1). [file 12902_2024_1545_MOESM1_ESM.docx]

**Appendices**

**Appendix 1. Search strategy**

**Appendix 2. The kappa score between the researchers**

**Appendix 3. PRISMA checklist**

**Appendix 4. The quality of evidence using the GRADE framework**

**Appendix 5. Assessment of study quality**

**Appendix 6. Funnel plot of meta-analysis of response rate (By R language 4.2.1)**

**Appendix 7. Forest plot of meta-analysis of response rate using the “cut-and-fill method” (By R language 4.2.1)**

**Appendix 1. Search strategy**

**Search strategy of CNKI**

The details of the search strategies (Dec 1, 2023)

(SU%= Graves‘ ophthalmopathy OR (TKA= thyroid associated ophthalmopathy OR TKA= dysthyroid ophthalmopathy OR TKA= Graves’ orbitopathy OR TKA=Graves’ orbitopathy OR TKA=myopathic ophthalmopathy OR TKA=infiltrative ophthalmopathy OR TKA=congestive ophthalmopathy OR TKA=edematous ophthalmopathy )) AND（SU%= glucocorticoids OR TKA= prednisone OR TKA= dexamethasone OR TKA= methylprednisolone）AND SU%=cyclophosphamide

**Search strategy of VIP**

The details of the search strategies (Dec 1, 2023)

M=(Graves‘ ophthalmopathy OR thyroid associated ophthalmopathy OR dysthyroid ophthalmopathy OR Graves’ orbitopathy OR myopathic ophthalmopathy OR infiltrative ophthalmopathy OR congestive ophthalmopathy OR edematous ophthalmopathy) AND M=(cyclophosphamide) AND M=(glucocorticoids)

**Search strategy of WanFang**

The details of the search strategies (Dec 1, 2023)

(Mesh: Graves‘ ophthalmopathy or (title/abstract: Graves‘ ophthalmopathy or thyroid associated ophthalmopathy or dysthyroid ophthalmopathy or Graves’ orbitopathy or myopathic ophthalmopathy or infiltrative ophthalmopathy or congestive ophthalmopathy or edematous ophthalmopathy)) and Mesh: glucocorticoids and cyclophosphamide

**Search strategy of SinoMed**

The details of the search strategies (Dec 1, 2023)

(Graves‘ ophthalmopathy[Title/Abstract] OR thyroid associated ophthalmopathy[Title/Abstract] OR dysthyroid ophthalmopathy[Title/Abstract] OR Graves’ orbitopathy[Title/Abstract] OR myopathic ophthalmopathy[Title/Abstract] OR infiltrative ophthalmopathy[Title/Abstract] OR congestive ophthalmopathy[Title/Abstract] OR edematous ophthalmopathy) [Title/Abstract]) AND (glucocorticoids[Title/Abstract] OR prednisone[Title/Abstract] OR dexamethasone[Title/Abstract] OR methylprednisolone[Title/Abstract] ) AND cyclophosphamide[Title/Abstract]

**Search strategy of Embase**

The details of the search strategies (Dec 1, 2023)

('endocrine ophthalmopathy'/exp OR 'endocrine ophthalmopathy' OR 'graves eye disease':ti,ab OR 'graves ophthalmopathy':ti,ab OR 'graves orbitopathy':ti,ab OR 'dysthyroid ophthalmopathy':ti,ab OR 'endocrine ophthalmopathy':ti,ab OR 'endocrine orbitopathy':ti,ab OR 'ophthalmopathy, endocrine':ti,ab OR 'thyroid associated eye disease':ti,ab OR 'thyroid associated ophthalmopathy':ti,ab OR 'thyroid associated orbitopathy':ti,ab OR 'thyroid eye disease':ti,ab OR 'thyroid ophthalmopathy':ti,ab OR 'thyroid orbitopathy':ti,ab) AND ('cyclophosphamide'/exp OR cyclophosphamide) AND ('glucocorticoid'/exp OR glucocorticoid OR 'glucocorticoid' OR 'glucocorticoid drug' OR 'glucocorticoid hormone' OR 'glucocorticoid steroid' OR 'glucocorticoids' OR 'glucocorticoids, synthetic' OR 'glucocorticoids, topical' OR 'glucocorticoidsteroid' OR 'glucocorticosteroid' OR 'glucocortoid' OR 'glycocorticoid' OR 'glycocorticosteroid')

**Search strategy of Pubmed**

The details of the search strategies (Dec 1, 2023)

#1 "Graves Ophthalmopathy"[Mesh]

#2 (((((((((((((((((((((((((((((((Ophthalmopathy, Graves[Title/Abstract]) OR (Ophthalmopathies, Thyroid-Associated[Title/Abstract])) OR (Thyroid-Associated Ophthalmopathies[Title/Abstract])) OR (Thyroid Associated Ophthalmopathies[Title/Abstract])) OR (Thyroid Eye Disease[Title/Abstract])) OR (Disease, Thyroid Eye[Title/Abstract])) OR (Eye Disease, Thyroid[Title/Abstract])) OR (Thyroid Eye Diseases[Title/Abstract])) OR (Thyroid-Associated Ophthalmopathy[Title/Abstract])) OR (Thyroid Associated Ophthalmopathy[Title/Abstract])) OR (Dysthyroid Ophthalmopathy[Title/Abstract])) OR (Dysthyroid Ophthalmopathies[Title/Abstract])) OR (Ophthalmopathy, Dysthyroid[Title/Abstract])) OR (Graves Eye Disease[Title/Abstract])) OR (Disease, Graves Eye[Title/Abstract])) OR (Eye Disease, Graves[Title/Abstract])) OR (Graves Orbitopathy[Title/Abstract])) OR (Orbitopathy, Graves[Title/Abstract])) OR (Ophthalmopathy, Thyroid-Associated[Title/Abstract])) OR (Ophthalmopathy, Thyroid Associated[Title/Abstract])) OR (Myopathic Ophthalmopathy[Title/Abstract])) OR (Myopathic Ophthalmopathies[Title/Abstract])) OR (Ophthalmopathy, Myopathic[Title/Abstract])) OR (Congestive Ophthalmopathy[Title/Abstract])) OR (Congestive Ophthalmopathies[Title/Abstract])) OR (Ophthalmopathy, Congestive[Title/Abstract])) OR (Edematous Ophthalmopathy[Title/Abstract])) OR (Edematous Ophthalmopathies[Title/Abstract])) OR (Ophthalmopathy, Edematous[Title/Abstract])) OR (Ophthalmopathy, Infiltrative[Title/Abstract])) OR (Infiltrative Ophthalmopathies[Title/Abstract])) OR (Infiltrative Ophthalmopathy[Title/Abstract])

#3 (#1 OR #2)

#4 "Glucocorticoids"[Mesh]

#5 ((((Glucocorticoid[Title/Abstract]) OR (Glucocorticoid Effect[Title/Abstract])) OR (Effect, Glucocorticoid[Title/Abstract])) OR (Glucorticoid Effects[Title/Abstract])) OR (Effects, Glucorticoid[Title/Abstract])

#6 (#4 OR #5)

#7 "Cyclophosphamide"[Mesh]

#8 (#3 AND #6 AND #7)

**Search strategy of Cochrane Library**

The details of the search strategies (Dec 1, 2023)

#1 MeSH descriptor: [Graves Ophthalmopathy] explode all trees

#2 ((“thyroid-associated ophthalmopathy” OR “thyroid eye disease” OR “Graves’ ophthalmopathy” OR “Graves’ orbitopathy” OR “endocrine ophthalmopathy” OR “endocrine orbitopathy” OR “thyroid associated orbitopathy”)):ti,ab,kw

#3 MeSH descriptor: [Glucocorticoids] explode all trees

#4 ((“Glucocorticoid Effect” OR “Effect, Glucocorticoid” OR “Glucocorticoid”)):ti,ab,kw

#5 MeSH descriptor: [Cyclophosphamide] explode all trees

#6 (#1 OR #2)

#7 (#3 OR #4)

#8 (#1 OR #2) AND (#3 OR #4) AND #5

**Search strategy of Cochrane Library**

The details of the search strategies (Dec 1, 2023)

(Graves Ophthalmopathy OR Thyroid Associated Ophthalmopathy OR Dysthyroid Ophthalmopathy OR Graves Orbitopathy OR Myopathic Ophthalmopathy OR Infiltrative Ophthalmopathy OR Congestive Ophthalmopathy OR Edematous Ophthalmopathy) AND (Cyclophosphamide) AND (Glucocorticoids)

**Appendix 2.** **The kappa score between the researchers**

The kappa score between QX and MY

| **QX * MY Crosstabulation** | | | | |
| --- | --- | --- | --- | --- |
| Count | | | | |
|  | | MY | | Total |
|  |  | exclusion | inclusion |  |
| QX | exclusion | 619 | 5 | 624 |
|  | inclusion | 7 | 38 | 45 |
| Total | | 626 | 43 | 669 |

| **Symmetric Measures** | | | | | |
| --- | --- | --- | --- | --- | --- |
|  | | Value | Asymptotic Standard Error^a^ | Approximate T^b^ | Approximate Significance |
| Measure of Agreement | Kappa | .854 | .041 | 22.096 | .000 |
| N of Valid Cases | | 669 |  |  |  |
| a. Not assuming the null hypothesis. | | | | | |
| b. Using the asymptotic standard error assuming the null hypothesis. | | | | | |

The kappa score between QX and MY

| **YC * SS Crosstabulation** | | | | |
| --- | --- | --- | --- | --- |
| Count | | | | |
|  | | SS | | Total |
|  |  | exclusion | inclusion |  |
| YC | exclusion | 27 | 2 | 29 |
|  | inclusion | 1 | 12 | 13 |
| Total | | 28 | 14 | 42 |

| **Symmetric Measures** | | | | | |
| --- | --- | --- | --- | --- | --- |
|  | | Value | Asymptotic Standard Error^a^ | Approximate T^b^ | Approximate Significance |
| Measure of Agreement | Kappa | .836 | .091 | 5.428 | .000 |
| N of Valid Cases | | 42 |  |  |  |
| a. Not assuming the null hypothesis. | | | | | |
| b. Using the asymptotic standard error assuming the null hypothesis. | | | | | |

**Appendix 3.** PRISMA checklist

| **Section and Topic** | **Item #** | **Checklist item** | **Reported on page #** |
| --- | --- | --- | --- |
| **TITLE** | | |  |
| Title | 1 | Identify the report as a systematic review. | Title Page |
| **ABSTRACT** | | |  |
| Abstract | 2 | See the PRISMA 2020 for Abstracts checklist. | 2 |
| **INTRODUCTION** | | |  |
| Rationale | 3 | Describe the rationale for the review in the context of existing knowledge. | 2 |
| Objectives | 4 | Provide an explicit statement of the objective(s) or question(s) the review addresses. | 2 |
| **METHODS** | | |  |
| Eligibility criteria | 5 | Specify the inclusion and exclusion criteria for the review and how studies were grouped for the syntheses. | 3 |
| Information sources | 6 | Specify all databases, registers, websites, organisations, reference lists and other sources searched or consulted to identify studies. Specify the date when each source was last searched or consulted. | 3 |
| Search strategy | 7 | Present the full search strategies for all databases, registers and websites, including any filters and limits used. | Supplementary Appendix 1 |
| Selection process | 8 | Specify the methods used to decide whether a study met the inclusion criteria of the review, including how many reviewers screened each record and each report retrieved, whether they worked independently, and if applicable, details of automation tools used in the process. | 3 |
| Data collection process | 9 | Specify the methods used to collect data from reports, including how many reviewers collected data from each report, whether they worked independently, any processes for obtaining or confirming data from study investigators, and if applicable, details of automation tools used in the process. | 3 |
| Data items | 10a | List and define all outcomes for which data were sought. Specify whether all results that were compatible with each outcome domain in each study were sought (e.g. for all measures, time points, analyses), and if not, the methods used to decide which results to collect. | 3 |
|  | 10b | List and define all other variables for which data were sought (e.g. participant and intervention characteristics, funding sources). Describe any assumptions made about any missing or unclear information. | 3 |
| Study risk of bias assessment | 11 | Specify the methods used to assess risk of bias in the included studies, including details of the tool(s) used, how many reviewers assessed each study and whether they worked independently, and if applicable, details of automation tools used in the process. | 3 |
| Effect measures | 12 | Specify for each outcome the effect measure(s) (e.g. risk ratio, mean difference) used in the synthesis or presentation of results. | 3 |
| Synthesis methods | 13a | Describe the processes used to decide which studies were eligible for each synthesis (e.g. tabulating the study intervention characteristics and comparing against the planned groups for each synthesis (item #5)). | 3 |
|  | 13b | Describe any methods required to prepare the data for presentation or synthesis, such as handling of missing summary statistics, or data conversions. | 3 |
|  | 13c | Describe any methods used to tabulate or visually display results of individual studies and syntheses. | 3 |
|  | 13d | Describe any methods used to synthesize results and provide a rationale for the choice(s). If meta-analysis was performed, describe the model(s), method(s) to identify the presence and extent of statistical heterogeneity, and software package(s) used. | 3 |
|  | 13e | Describe any methods used to explore possible causes of heterogeneity among study results (e.g. subgroup analysis, meta-regression). | 3 |
|  | 13f | Describe any sensitivity analyses conducted to assess robustness of the synthesized results. | 3 |
| Reporting bias assessment | 14 | Describe any methods used to assess risk of bias due to missing results in a synthesis (arising from reporting biases). | 3 |
| Certainty assessment | 15 | Describe any methods used to assess certainty (or confidence) in the body of evidence for an outcome. | 3 |
| **RESULTS** | | |  |
| Study selection | 16a | Describe the results of the search and selection process, from the number of records identified in the search to the number of studies included in the review, ideally using a flow diagram. | Figure 1 |
|  | 16b | Cite studies that might appear to meet the inclusion criteria, but which were excluded, and explain why they were excluded. | Figure 1 |
| Study characteristics | 17 | Cite each included study and present its characteristics. | Table 1 |
| Risk of bias in studies | 18 | Present assessments of risk of bias for each included study. | Figure 2 |
| Results of individual studies | 19 | For all outcomes, present, for each study: (a) summary statistics for each group (where appropriate) and (b) an effect estimate and its precision (e.g. confidence/credible interval), ideally using structured tables or plots. | Figure 3 |
| Results of syntheses | 20a | For each synthesis, briefly summarise the characteristics and risk of bias among contributing studies. | 4 |
|  | 20b | Present results of all statistical syntheses conducted. If meta-analysis was done, present for each the summary estimate and its precision (e.g. confidence/credible interval) and measures of statistical heterogeneity. If comparing groups, describe the direction of the effect. | 4 |
|  | 20c | Present results of all investigations of possible causes of heterogeneity among study results. | 4 |
|  | 20d | Present results of all sensitivity analyses conducted to assess the robustness of the synthesized results. | 4 |
| Reporting biases | 21 | Present assessments of risk of bias due to missing results (arising from reporting biases) for each synthesis assessed. | 4 |
| Certainty of evidence | 22 | Present assessments of certainty (or confidence) in the body of evidence for each outcome assessed. | Supplementary Appendix 4 |
| **DISCUSSION** | | |  |
| Discussion | 23a | Provide a general interpretation of the results in the context of other evidence. | 4, 5 |
|  | 23b | Discuss any limitations of the evidence included in the review. | 5 |
|  | 23c | Discuss any limitations of the review processes used. | 5 |
|  | 23d | Discuss implications of the results for practice, policy, and future research. | 5 |
| **OTHER INFORMATION** | | |  |
| Registration and protocol | 24a | Provide registration information for the review, including register name and registration number, or state that the review was not registered. | N/A |
|  | 24b | Indicate where the review protocol can be accessed, or state that a protocol was not prepared. | N/A |
|  | 24c | Describe and explain any amendments to information provided at registration or in the protocol. | N/A |
| Support | 25 | Describe sources of financial or non-financial support for the review, and the role of the funders or sponsors in the review. | 6 |
| Competing interests | 26 | Declare any competing interests of review authors. | 6 |
| Availability of data, code and other materials | 27 | Report which of the following are publicly available and where they can be found: template data collection forms; data extracted from included studies; data used for all analyses; analytic code; any other materials used in the review. | 6 |

*From:*  Page MJ, McKenzie JE, Bossuyt PM, Boutron I, Hoffmann TC, Mulrow CD, et al. The PRISMA 2020 statement: an updated guideline for reporting systematic reviews. BMJ 2021;372:n71. doi: 10.1136/bmj.n71

For more information, visit: <http://www.prisma-statement.org/>

**Appendix 4. The quality of evidence using the GRADE framework**

| **Certainty assessment** | | | | | | | **№ of patients** | | **Effect** | | **Certainty** | **Importance** |
| --- | --- | --- | --- | --- | --- | --- | --- | --- | --- | --- | --- | --- |
| **№ of studies** | **Study design** | **Risk of bias** | **Inconsistency** | **Indirectness** | **Imprecision** | **Other considerations** | **CYC/GCs** | **GCs** | **Relative (95% CI)** | **Absolute (95% CI)** |  |  |
| **CYC/GCs vs GCs** | | | | | | | | | | | | |
| 6 | randomised trials | not serious | not serious | not serious | not serious^a^ | publication bias strongly suspected^b^ | 199/247 (80.6%) | 152/239 (63.6%) | **RR 1.12** (1.07 to 1.17) | **76 more per 1,000** (from 45 more to 108 more) | ⨁⨁⨁◯ Moderate | CRITICAL |
| **CYC/GCs vs negative control** | | | | | | | | | | | | |
| 6 | randomised trials | not serious | not serious | not serious | not serious^a^ | publication bias strongly suspected^b^ | 185/203 (91.1%) | 113/199 (56.8%) | **RR 1.15** (1.11 to 1.20) | **85 more per 1,000** (from 62 more to 114 more) | ⨁⨁⨁◯ Moderate | CRITICAL |

CI: confidence interval; RR: risk ratio

a. because data for some of the continuous variables were incomplete, we were forced to complete our data analyses only using the dichotomous variables, limiting precision.

b. Egger test (t = 3.94, P = 0.0023) suggested a risk of publication bias

**Appendix 5.** Assessment of study quality

| **Study** | **Random sequence generation** | **Allocation concealment** | **Blinding of participants and personnel** | **Incomplete outcome data** | **Selective reporting** | **Other bias** |  |  |  |  |
| --- | --- | --- | --- | --- | --- | --- | --- | --- | --- | --- |
| Shi 2004[23] | Unclear risk | Unclear risk | Unclear risk | Low risk | Low risk | Unclear risk |  |  |  |  |
| Liu 2005[24] | High risk | Unclear risk | Unclear risk | Low risk | Low risk | Unclear risk |  |  |  |  |
| Zhang 2005[22] | Unclear risk | Unclear risk | High risk | Low risk | Low risk | Unclear risk |  |  |  |  |
| Ruan 2006[25] | Unclear risk | Unclear risk | Unclear risk | Low risk | Low risk | Unclear risk |  |  |  |  |
| Tang 2006[26] | Unclear risk | Unclear risk | Unclear risk | Low risk | Low risk | Unclear risk |  |  |  |  |
| Su 2014[27] | Low risk | Unclear risk | Unclear risk | Low risk | Low risk | Unclear risk |  |  |  |  |
| Dai 2015[28] | Unclear risk | Unclear risk | Unclear risk | Low risk | Low risk | Unclear risk |  |  |  |  |
| Wang 2015[29] | High risk | Unclear risk | Unclear risk | Low risk | Low risk | Unclear risk |  |  |  |  |
| Wen 2016[30] | Unclear risk | Unclear risk | High risk | Low risk | Low risk | Unclear risk |  |  |  |  |
| Gao 2018[31] | Low risk | Unclear risk | Unclear risk | Low risk | Low risk | Unclear risk |  |  |  |  |
| Liang 2020[32] | Low risk | Unclear risk | Unclear risk | Low risk | Low risk | Unclear risk |  |  |  |  |
| Cajal 2020[33] | High risk | Unclear risk | Unclear risk | Low risk | Low risk | Unclear risk |  |  |  |  |
| Wang 2021[34] | Low risk | Unclear risk | Unclear risk | Low risk | Low risk | Unclear risk |  |  |  |  |

**Appendix 6.** Funnel plot of meta-analysis of response rate (By R language 4.2.1)

0.5

1.0

2.0

5.0

10.0

0.6

0.5

0.4

0.3

0.2

0.1

0.0

Standard Error


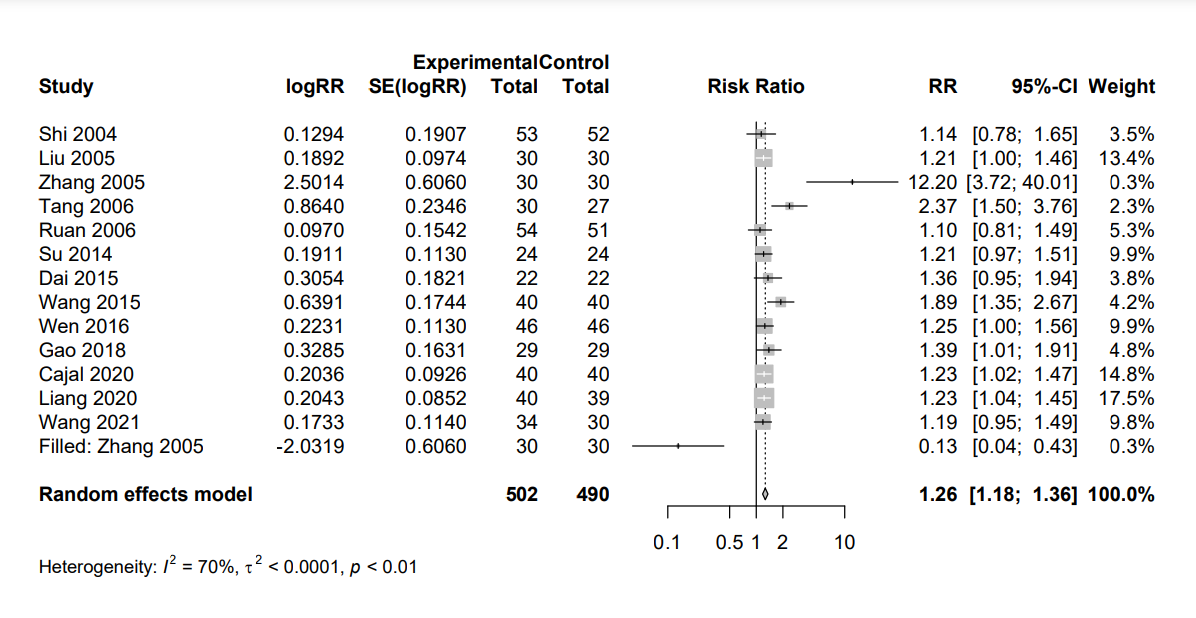
**Appendix 7. Forest plot of meta-analysis of response rate using the “cut-and-fill method” (By R language 4.2.1)**
